# Neonatal encephalopathy multiorgan scoring systems: systematic review

**DOI:** 10.3389/fped.2024.1427516

**Published:** 2024-10-01

**Authors:** Noor Adeebah Mohamed Razif, Aidan D’Arcy, Sarah Waicus, Alyssa Agostinis, Rachelle Scheepers, Yvonne Buttle, Aidan Pepper, Aisling Hughes, Basem Fouda, Panya Matreja, Emily MacInnis, Mary O’Dea, Eman Isweisi, Philip Stewart, Aoife Branagan, Edna F. Roche, Judith Meehan, Eleanor J. Molloy

**Affiliations:** ^1^ Discipline of Pediatrics, Trinity College Dublin, The University of Dublin, Dublin, Ireland; ^2^ TrinityTranslational Medicine Institute (TTMI), St James Hospital, Dublin, Ireland; ^3^ Trinity Research in Childhood Centre (TRiCC), Trinity College Dublin, Dublin, Ireland; ^4^ Pediatrics, Coombe Hospital, Dublin, Ireland; ^5^ Neonatology, Children's Health Ireland, Dublin, Ireland; ^6^ Endocrinology, Children's Health Ireland (CHI) at Tallaght, Dublin, Ireland; ^7^ Neurodisability, Children's Health Ireland (CHI) at Tallaght, Dublin, Ireland

**Keywords:** neonatal encephalopathy, therapeutic hypothermia, brain injury, multiorgan dysfunction score, scoring system

## Abstract

**Introduction:**

Neonatal encephalopathy (NE) is a condition with multifactorial etiology that causes multiorgan injury to neonates. The severity of multiorgan dysfunction (MOD) in NE varies, with therapeutic hypothermia (TH) as the standard of care. The aim is to identify current approaches used to assess and determine an optimum scoring system for MOD in NE.

**Methods:**

The systematic review conformed to the Preferred Reporting Items for Systematic Reviews and Meta-Analyses (PRISMA) guidelines. An electronic search was conducted using PubMed, EMBASE, MEDLINE, Cochrane Central Register of Controlled Trials, Scopus, and CINAHL for studies of scoring systems for MOD in NE.

**Results:**

The search yielded 628 articles of which 12 studies were included for data extraction and analysis. Five studies found a positive correlation between the severity of NE and MOD. There was significant heterogeneity across the scoring systems, including the eligibility criteria for participants, the methods assessing specific organ systems, the length of follow-up, and adverse outcomes. The neurological, hepatic, cardiovascular, respiratory, hematological, and renal systems were included in most studies while the gastrointestinal system was only in three studies. The definitions for hepatic, renal, and respiratory systems dysfunction were most consistent while the cardiovascular system varied the most.

**Discussion:**

A NE multiorgan scoring system should ideally include the renal, hepatic, respiratory, neurological, hematological, and cardiovascular systems. Despite the heterogeneity between the studies, these provide potential candidates for the standardization of MOD scoring systems in NE. Validation is needed for the parameters with adequate length of follow-up beyond the neonatal period. Additionally, the evaluation of MOD may be affected by TH considering its multiorgan effects.

## Introduction

Neonatal encephalopathy (NE) has been described as a “clinically defined syndrome of disturbed neurological function in the earliest days of life in the term infant, manifested by difficulty with initiating and maintaining respiration, depression of tone and reflexes, subnormal level of consciousness, and often by seizures” (1). This definition of NE has been updated to include late preterm infants born at 35–36 weeks (2).

NE is a condition with diverse etiological factors that are present either alone or in combination (3). These causes include infection, coagulopathy, inborn errors of metabolism, placental pathology, and hypoxia-ischemia (3). Despite the diverse aetiology of NE, in over half of the cases, the cause is not determined (1). Hypoxic ischemic encephalopathy (HIE) has been defined by the American College of Obstetricians and Gynecologists (ACOG) and the American Academy of Pediatrics (AAP) as a subtype of NE “for which the aetiology is considered to be limitation of oxygen and blood flow near the time of birth” (2). In summary, NE is an “umbrella term”, which does not specify the aetiology of the neonate's condition (4).

HIE is a term often used interchangeably with NE (5). It has been noted that some clinicians use the term HIE prior to confirmation of hypoxia-ischemia being the cause of NE at birth (6). Perinatal asphyxia is another term frequently encountered in our review of the literature pertaining to NE. The ACOG and AAP note that there is a variation among studies in their definitions of asphyxia and use the term to describe “marked impairment of gas exchange leading, if prolonged, to progressive hypoxemia, hypercapnia and significant metabolic acidosis” (2). The misuse of the labels HIE and perinatal asphyxia may incorrectly suggest other determinants have no role in the pathogenesis of NE. Therefore, in our paper we will use the term NE to include HIE and NE secondary to other causes.

Neurological dysfunction is central to the clinical manifestations of NE and is the most accurate predictor of long term outcomes, however, infants with NE can also experience extra-neurological injury (7). Non-neurological organ dysfunction has been observed to have negative effects on long term outcomes and acute treatment efficacy. NE is associated with increased risk of multiorgan dysfunction (MOD). One mechanism is secondary to global hypoxia following interruption of the placental blood supply (8). The multi-etiological component of NE also means that metabolic, infectious, placental dysfunction, and genetic factors are all likely to play a role in the MOD seen in NE. MOD primarily affects the cardiovascular, respiratory, immune, renal, endocrine, and hepatic systems via various clinical, radiological, or biochemical parameters (7). Despite documented descriptions of MOD syndrome in NE, there are no standardized consensus definitions of individual organ dysfunction (7).

The extent and severity of MOD in NE can vary depending on the underlying cause and severity of the condition. In some cases, infants may experience mild MOD, while in other cases, the degree of dysfunction may be life-threatening. No standard scoring system currently exists to predict the severity of MOD in NE, although some such as the Multiorgan dysfunction scoring in neonatal encephalopathy (MODE Score) have been suggested (9) (Table 1).

**Table 1:** Comparison of included studies on multiorgan dysfunction in NE.

	Number of patients; male sex (%)	Gestational age (weeks)	Birth weight (g)	Apgar score at 5 min	Umbilical cord artery or blood gas pH	Length of follow-up days	Inclusion criteria
Perlman et al., USA (10)	35; N/A	40 ± 1.9	3193 ± 768	≤5 (77.2%) 6 (17.1%) ≥7 (5.7%)	7.11 ± 0.14	4	Asphyxia defined as: • 5 min Apgar score of ≤5 in initially non-intubated or intubated infants • 5 min Apgar score of 6 in intubated infants • Umbilical cord arterial pH of <7.20, or an umbilical cord arterial PCO_2_ > 50 mmHg
Martin-Ancel et al., Spain (11)	72; 52.8	N/A	3215 ± 490	≥7 (46%) 4–6 (37%) ≤3 (14%)	7.04 ± 0.13	7	Perinatal asphyxia if: Fetal scalp blood pH <7.20 • Umbilical cord arterial pH <7.20 • Apgar scores <4 at 1 min and/or <7 at 5 min • Requirement of >1 min of positive pressure ventilation before sustained respiration occurred
Hankins et al., USA (12)	46; N/A	N/A	N/A	<6 (83%)	<7.0 (67%)	5	Acute intrapartum hypoxic event if: • A sentinel (signal) hypoxic event occurring during, or immediately preceding labor, such as placental abruption or an umbilical cord prolapse • A sudden, rapid, and sustained deterioration of the fetal heart rate pattern, usually after the hypoxic sentinel event where the pattern was previously normal, or an obvious and acute catastrophic event evident immediately upon presentation, • Early onset of NE in infants ≥32 weeks gestational age, and • Absence of a severe congenital abnormality that would impact transition from fetal to neonatal life
Shah et al., Canada (8)	130 of 144 with outcome data; N/A	Good outcome: 40.2 ± 1.6 Adverse outcome: 39.9 ± 1.6	Good outcome: 3488 ± 509 Adverse outcome: 3420 ± 500	Good outcome: 4^a^ Adverse outcome: 3^a^	N/A	24 months	Post-intrapartum asphyxial HIE: • One or more of following: 5 min Apgar score <5, metabolic acidosis (cord arterial blood or blood gas analysis within first hour after birth) indicated by a base deficit ≥16 mmol/L, or delayed onset of respiration for ≥5 min • Need for mechanical ventilation at birth • Evidence of encephalopathy (altered state of consciousness and/or seizures using Volpe's criteria) • Infants who had complete clinical and/or investigational assessments of the function of all four organs as outlined in the study
Tarcan et al., Turkey (13)	ALT <100 U/L: 34; 82.4 ALT >100 U/L: 22; 68.2	ALT <100 U/L: 37.8 ± 2.6 ALT >100 U/L: 38.1 ± 2/7	ALT <100U/L: 2873 ± 724 ALT >100 U/L: 2993 ± 722	ALT <100 U/L: 6.1 ± 2.4 (*n* = 26) ALT >100U/L: 5.2 ± 2 (*n* = 16)	N/A	N/A	Perinatal asphyxia if: • 5 min Apgar score <5 or cord blood pH <7.10 • Clinical signs of encephalopathy according to the Sarnat score • Multiorgan involvement (brain and at least one other organ)
Basu et al. UK, USA, New Zealand (14)	194; 53	39.1 ± 1.5	3470 ± 651	2.0^a^ (0–4.0)	6.87 ± 0.23	18 months	• ≥36 weeks of gestation with moderate-to-severe HIE based on Sarnat criteria • Apgar score of ≤5 at 10 min after birth • A continued need for resuscitation, including endotracheal or mask ventilation at 10 min after birth • Severe acidosis (pH <7.00 or a base deficit of ≥16 mmol/L in an umbilical cord blood sample or an arterial or venous blood sample obtained within 60 min of birth)
Thorsen et al. Netherlands (15)	142; 60	40^a^	3362 ± 605	N/A	6.97 ± 0.19	N/A	• Gestational age >36 weeks • Suffering perinatal asphyxia (i.e., ongoing resuscitation at 10 min after birth, cord-blood or 1 h postnatal blood gas analysis indicating pH <7.0 or base deficit >16 mmol/L) with clinical signs of moderate-to-severe encephalopathy • Available postnatal Thompson encephalopathy score assessment and undergoing neuroprotective treatment by therapeutic hypothermia <6 h postnatal in neonatal intensive care setting
Alsina et al., Spain (16)	79; 69	38.8 ± 1.8	3170 ± 570	5^a^ (3–7)	6.94 ± 0.19	3 days	HIE within 6 h of life if: • NE, defined as a syndrome of neurologic dysfunction manifested by a subnormal level of consciousness with or without seizures or palmar hyperexcitability (tremor, overactive myotatic reflexes, hypersensitivity to stimulation, or startle responses) • At least one of the following clinical surrogates of hypoxic-ischemic insult: altered fetal heart rate pattern, sentinel event, labor dystocia, Apgar ≤5 at 5 min, or acidosis at birth (pH ≤7.0 in arterial umbilical cord)
Michniewicz et al., Poland (17)	Group A (Sarnat stage 2): 27; 55.56 Group B (Sarnat stage 3): 30; 46.67	Group A: 39.22 ± 1.91 Group B: 38.33 ± 1.56	Group A: 3314.26 ± 507.79 Group B: 3306.16 ± 641.89	Group A: 4^a^ (3–6) Group B: 3^a^ (0–5)	Group A: 7.10 ± 0.18 Group B: 2.5 ± 0.22	48 h	• Term newborns diagnosed with HIE • Met local criteria for therapeutic hypothermia (not detailed)
Sweetman et al., Ireland (9)	85; N/A	N/A	N/A	N/A	N/A	12–24 months	• >36 weeks gestation, admitted to NICU • Fulfilled the Huang criteria for perinatal asphyxia requiring resuscitation at birth: ○ Abnormal neurological signs, and/or other organ dysfunction ○ At least two of the following three criteria: (a) evidence or suspicion of hypoxic-ischemic injury based on a history of fetal distress, (b) need for resuscitation after birth, (c) base deficit of >14 mmol/L or pH <7.2 in cord blood or admission arterial blood gas
Terzic et al., Bosnia and Herzegovina (18)	91; 58.3	39.15 ± 1.88	3404.8 ± 717.04	4.12 ± 1.65	6.99 ± 0.18	72 h after starting therapeutic hypothermia	• Sarnat stage 2 and 3 • Minimal gestational age of 36 weeks • Minimal birth weight of 2,500 g • Maximal postnatal age 6 h • Apgar score 10 min after birth ≤5, or need for resuscitation 10 min after birth, or pH <7.00, or base excess ≥ −16 mmol/L within 60 min after birth
Yan et al., USA (19)	157; 62.4	39.0 ± 1.6	3287 ± 639	4^a^ (2–5)	Arterial cord pH: 6.94 ± 0.21 Initial blood gas: 7.07 ± 0.19	3 days of life	• ≥36 weeks gestation who received therapeutic hypothermia • Modified Sarnat score with ≥3 categories with moderate or severe findings • Neonates with seizures in the first 6 h after birth did not need to meet the modified Sarnat score • pH of ≤7.0 or less or a base deficit of 16 mmol/L or more in a sample of umbilical-cord blood or any blood during the first hour after birth • Additional criteria (if biochemical criteria not met): an acute perinatal event (e.g., late or variable decelerations, cord prolapse, cord rupture, uterine rupture, maternal trauma, hemorrhage, or cardiorespiratory arrest) and either a 10 min Apgar score of ≤5 or assisted ventilation initiated at birth and continued for ≥10 min

N/A, not assessed; hrs, hours; EMCS, emergency Cesarean section; SVD, spontaneous vaginal delivery; ELSCS, elective lower section Cesarean section; CS, Cesarean section; PCO_2_, partial pressure of carbon dioxide; NICU, neonatal intensive care unit; HIE, hypoxic-ischemic encephalopathy; NE, neonatal encephalopathy; ALT, alanine transaminase.

Values are typically presented as mean ± SD unless stated otherwise.

^a^ Indicates median (interquartile range).

Several single organ scoring systems are currently used in the assessment of NE, including the Sarnat and modified Sarnat scores, and severity prediction based on imaging or blood tests. Single organ systems such as the Thompson score are associated with prediction of adverse neurodevelopmental outcome but are not associated with the degree of MOD (15).

To our knowledge, there has been no systematic review published that discusses the assessment of multiorgan dysfunction scoring systems in NE. The objective of this systematic review was to identify and summarize approaches used to score MOD in NE. We aimed to discuss the heterogeneity between the studies and offer a critical appraisal of MOD scoring in NE.

## Methods

### Information sources

The review was performed according to the PRISMA (Preferred Reporting Items for Systematic Reviews and Meta-Analyses) guidelines (20). Electronic databases including PubMed, EMBASE, MEDLINE, Cochrane Central Register of Controlled Trials, Scopus, and CINAHL were searched in February 2023. In addition to databases, grey literature was also searched from Google Scholar. There was no restriction on the publication date.

### Literature search strategy

The following keywords were used in the search of databases, registers, and grey literature (“hypoxia-ischaemia, brain” OR “newborn” OR “neonatal encephalopathy” OR “asphyxia neonatorum”) AND (“multiple organ failure” OR “multiple organ dysfunction” OR “Sarnat score” OR “Thompson encephalopathy score” OR “multiorgan dysfunction in neonatal encephalopathy scoring”).

### Eligibility criteria of studies for inclusion

The population included neonates aged <28 days old with HIE or NE. Adults, adolescents, and children >28 days old without HIE or NE were excluded. Scoring systems that evaluated the dysfunction of an organ or multiorgan system including hepatic, hematological, cardiac, respiratory, gastrointestinal, renal, reproduction, immune, endocrine, or a combination of these systems were included. Articles that did not report scoring systems, evaluated neurological dysfunction alone, or assessed organ dysfunction in unrelated conditions were excluded. Reported outcomes included the extent of organ injury along with prognosis, mortality, or prediction of severity and measures of reliability, validity, or accuracy. Measures not predicting the reliability, validity, or accuracy of scoring systems were excluded. Study designs included randomized controlled trials (RCTs), literature reviews, retrospective cohorts, prospective cohorts, observational studies, cross-sectional studies, meta-analyses, systematic reviews, or longitudinal series that are published in English. Non-controlled trials, commentary, experimental animal studies, or articles that were not published in English were excluded.

### Data extraction

**Figure 1:**
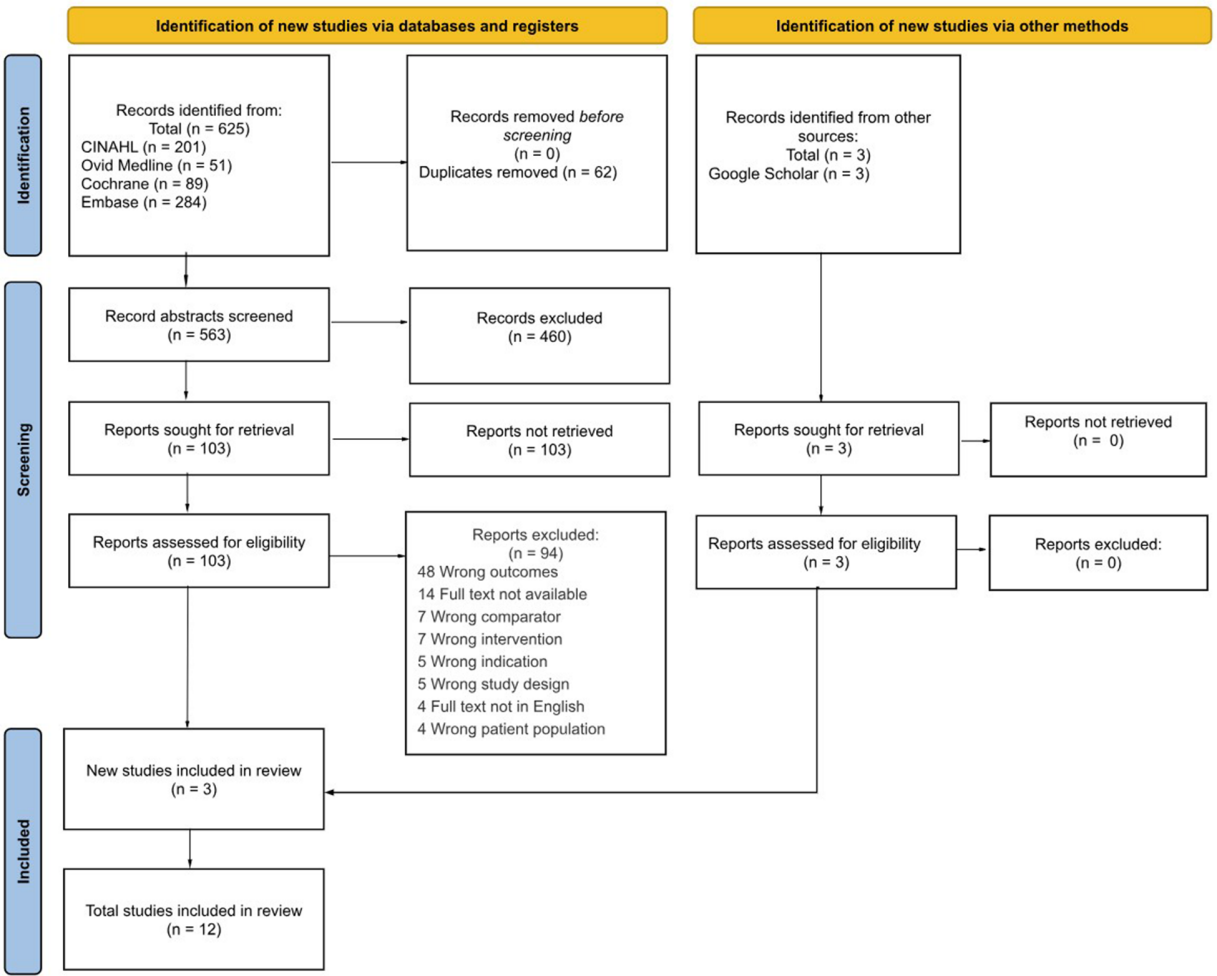
PRISMA flow diagram of study selection for multiorgan scoring systems in neonatal encephalopathy. An initial list of studies was first compiled using keyword search via databases and registers, (left) and other sources (21) to Covidence. Abstracts were initially screened for relevance, and then the studies were reviewed against the inclusion criteria. The final included studies that met the inclusion criteria were included in the review.

Each database and register were independently searched and title, authors, citation, publication year, type of study, language, scoring system, and abstract were extracted into Covidence for screening. After articles were compiled, duplicates between each database were removed before commencing the evaluation of the inclusion criteria. For each article appraisal, the title and abstract were first evaluated for inclusion, followed by a full-text review. Each article was independently screened by two reviewers, and disagreements were resolved by a third reviewer with consensus-based discussion. Inclusion or exclusion and reason were documented for each article on the excel sheet and can be seen in Figure 1. Characteristics of each of the included studies were extracted and summarized in Table 1.

### Outcome measures

The primary endpoint is to assess the differences in multiorgan systems used for the prediction of severity, mortality, and prognosis neonates with NE.

## Results

The search yielded 625 articles based on main search and 3 from Google Scholar, of which 62 were duplicates (Figure 1). The remaining 566 studies were screened for relevance which resulted in 106 remaining studies for full-text review. The 94 studies were excluded for various reasons. Therefore, 12 studies were included for data extraction and analysis.

Table 1 details the characteristics of each included study with the relevant parameters. Studies were mainly carried out in Europe and in North America. The total number of patients in the study ranged from 35 to 194. More male neonates were enrolled in the studies than female neonates. All studies enrolled term neonates with birth weight around 3,000 g, although some studies (9, 11, 12) did not specify the mean or median of the parameters. All studies specified the inclusion criteria to include term neonates that meet the diagnosis of NE based on clinical and biochemical findings. The total length of follow up on the patients ranged between 48 h and 24 months. A summary of variables used to assess MOD included neurological (clinical examination, cranial nerve imaging, EEG and presence of encephalopathy), cardiovascular (ECG, ECHO, CK-MB, BP, MAP, troponin T, pressors), respiratory (use of ventilators, oxygen support, presence of pulmonary hypertension), gastrointestinal (time to oral feed, presence of necrotising enterocolitis, and abdominal distension), hepatic (elevated aminotransferases, or lactate dehydrogenase), renal (serum creatinine, oligemia, or azotemia), and hematological (presence of polycythemia, normoblastemia, thrombocytopenia, or increased clotting time) variables.

**Figure 2:**
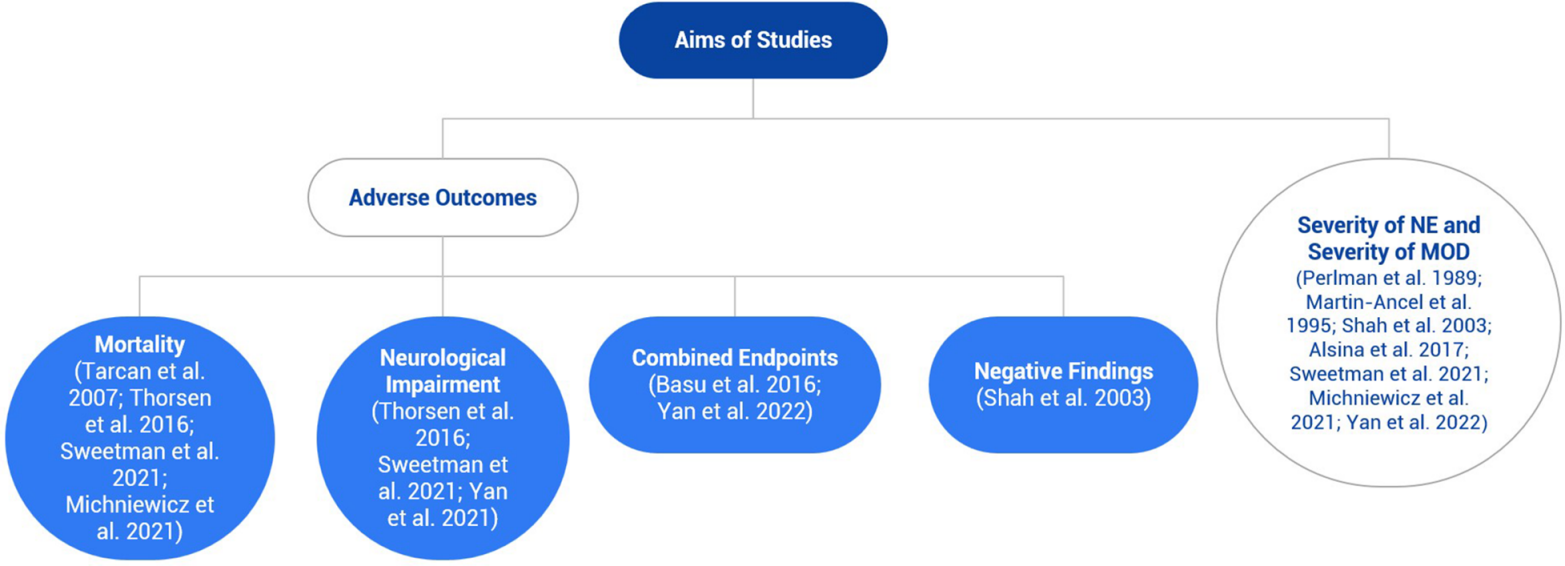
Flowchart illustrating the different aims of the included studies, which is mainly divided into adverse outcomes and the severity of neonatal encephalopathy (NE) and multiorgan dysfunction. Most studies investigated the latter aim, but some examined both aims.

The included studies had varying objectives- investigation of specific adverse outcomes or the relationship between the severity of NE and the severity of MOD (Figure 2).

### Studies focusing on adverse outcomes

Several of the included studies assessed whether multiorgan dysfunction scoring can be correlated with specific adverse outcomes.

#### Mortality

The MODE score assigns one point for every abnormality in cardiovascular (troponin-T > 0.1 ng/ml, heart rate of <80 bpm), respiratory (need for ventilation for >72 h or oxygen requirement >72 h), gastrointestinal (aspartate transaminase (AST) or alanine transaminase (ALT) > 100 IU/L), hematological (platelet count of <60 × 10^9^/L, prothrombin time (PT) > 20 s or fibrinogen level <1.0 g/L), and neurological (abnormal cranial US or MRI scan) organ systems with a maximum score of 15. Sweetman et al. found that amongst infants with perinatal asphyxia, the MODE score was highly correlated with mortality (9). Similarly, Michniewicz et al. studying infants with HIE, it was found that MOD was significantly correlated with infant death. This study defined MOD as dysfunction in ≥2 of the following organ systems; cardiovascular by evaluation of hemodynamics and contractility, renal by presence of serum creatinine levels >1.5 mg/dl and oliguria of <0.5 ml/kg/h, hepatic by increase in AST or ALT >100 IU/L, respiratory by presence of respiratory distress syndrome, meconium aspiration syndrome, or pneumonia, and bone marrow by measuring a platelet count <100,000/mm^3^. Interestingly, when kidney dysfunction and bone marrow insufficiency are assessed separately, they were also found to be significantly correlated with infant death (17).

Terzic et al. assessed outcomes for cooled newborns after perinatal asphyxia which included levels of pH, base excess (BE), lactate, AST, lactate dehydrogenase (LDH), activated partial thromboplastin time (APTT), and international normalized ratio (INR), and all were significantly higher in the non-survivor vs. survivor group (18). Tarcan et al. showed that in a group of infants with perinatal asphyxia, those who have hepatic involvement (defined as ALT > 100 U/L), had a significantly higher death (13). Thorsen et al. found that in a cohort of asphyxiated newborns treated with TH, a Thompson encephalopathy score ≥12 is associated with higher rates of death before discharge, compared to a score <7 which does not warrant hypothermia treatment protocol (15).

#### Neurological impairment

In addition to assessing correlation with mortality, several included studies assessed the ability of multi-system scoring systems to predict the occurrence of neurodevelopmental problems. Sweetman et al. found that higher MODE scores are associated with higher rates of abnormal neurological examination at discharge (9). Significant differences in MODE scores were also found between infants with normal vs. abnormal Bayley-III scores at 2 years for all three scoring categories (cognitive, language, and motor scores). Furthermore, a significant negative correlation was found between MODE scores and Bayley-III scores for the language and motor domains (9).

Some studies investigated the association with existing neurological scores. Thorsen et al. found that a Thompson encephalopathy score ≥12 was associated with higher rates of severe neonatal seizures among asphyxiated newborns treated with TH compared to scores between 7 and 11 (15). The Sarnat score was mainly used as the inclusion criteria to meet the aims of the studies (2, 13, 14, 18, 19). Michniewicz et al. divided the participants into stage II and stage III HIE based on the Sarnat score to assess mortality, however, neurological impairment was not included in the MOD criteria for the study outcome (17).

Yan et al. found that injuries to the basal ganglia and thalamus regions measured using magnetic resonance imaging (MRI) were strongly associated with respiratory dysfunction, whereas injuries to watershed regions were most strongly associated with hepatic dysfunction (19). Sixty-eight infants had an MRI scan in Sweetman et al., but only 31 of them reported abnormal results (9).

#### Combined endpoints

In the study by Basu et al. it was found that hypoglycemic and hyperglycemic infants with HIE had higher rates of death and/or severe neurological disabilities at 18 months compared to normoglycemic infants. Furthermore, it was found that the risk is higher for infants suffering from hypoglycemia than hyperglycemia. It is worth noting that the severity of MOD followed a similar pattern, with maximal severity occurring amongst hypoglycemic followed by hyperglycemic and normoglycemic infants (14).

Yan et al. studied the association between MOD and a combined primary endpoint of death or moderate-to-severe brain injury (19). A strong association was found between the number of organ systems affected and rates of adverse outcomes. Among the organ systems, neurological and renal dysfunction were most strongly correlated with the primary outcome. Interestingly, however, there was no correlation found in the case of pulmonary hypertension (19).

#### Negative findings

Contrary to the above papers, Shah et al. failed to find any relation between multiorgan involvement and long-term adverse outcomes in infants with post-asphyxial HIE (8). In this paper adverse outcomes were defined as either of the following: (1) Death due to post-asphyxial HIE, (2) Severe cerebral palsy by 12 months of age, (3) Mild or Moderate cerebral palsy with blindness or deafness by 12 months of age, or (4) Moderate cerebral palsy with suspected developmental delay at 12 months (confirmed by a Bayley score <2 standard deviation below the mean at 21–24 months). It is worthy of note however, that compared to the group with good outcomes, the group with adverse outcomes showed marginal differences in rates of kidney and cardiovascular system dysfunction, but no differences in pulmonary or hepatic dysfunction. Additionally, none of the infants received TH as it was not the standard of care at the time (8).

### Studies focusing on the relationship between severity of NE and severity of MOD

Nine of the twelve studies in this review sought to analyze the relationship between the severity of perinatal asphyxia and degree of MOD present in neonates. Shah et al., Sweetman et al., Michniewicz et al., Yan et al., and Alsina et al. all found a positive correlation between the severity of HIE, and both the number of organs involved and the degree of organ dysfunction (8, 9, 16, 17, 19). Martin-Ancel et al. also reported a positive relationship between perinatal asphyxia and severity of organ dysfunction but found that Apgar scores were the only biomarker of perinatal asphyxia they looked at that demonstrated this association (11). They did not find a positive correlation between umbilical cord arterial blood pH, meconium-stained amniotic fluid, umbilical cord abnormalities, presentation, or type of delivery and degree of organ dysfunction (11). Additionally, Hankins et al. reported that injury to the cardiac, central nervous, hematologic, hepatic, and/or renal systems were more likely to occur (12). Conversely, two studies found no relationship between the severity of perinatal asphyxia and degree of MOD. Thorsen et al. did not establish a correlation between any level of Thompson score and development of MOD (15). The study by Perlman et al. found that there was no relationship between obstetric variables, Apgar score, cord arterial blood partial pressure oxygen, partial pressure carbon dioxide, pH, and/or base excess, and organ dysfunction (10). They did, however, find that evidence of HIE was more common in both infants with prolonged low Apgar scores and in those requiring cardiopulmonary resuscitation (CPR) (10).

## Discussion

This systematic review was carried out to evaluate currently available scoring systems for MOD in NE. As previously discussed, scoring systems for the assessment of encephalopathy exist and are frequently used in NE, these include the Sarnat score (22), scores based on it (23), and the Thompson score (24). Although neurological dysfunction is one of the most obvious manifestations of NE, MOD is increasingly recognized as a major source of morbidity and mortality in NE (7). Despite this knowledge, a unified and validated scoring system is not currently in mainstream clinical or research use for assessing this dysfunction. Only Alsina et al. and Sweetman et al. devised a quantified scoring system based on cut off values of the variables measured (9, 16). The parameters identified in this review provide potential candidates for correcting the absence of a consensus MOD scoring system in NE, which may facilitate further research in this area. Developing an MOD scoring system would be beneficial in standardizing the determining the severity of NE, aid in prognosis, and inform organ-specific care to optimize outcomes for high-risk neonates in adverse outcomes.

This review identified 12 systems in the literature that assessed MOD in NE. Organ systems were assessed using combinations of clinical, radiological, and biochemical parameters. Though there was variation between systems relating to the combinations of organ systems assessed or the definition of organ dysfunction, similar components were observed throughout most systems evaluated. All twelve studies included some combination of at least three systems: neurological, cardiovascular, respiratory, gastrointestinal, hepatic, renal, or hematological dysfunction (Table 2).

**Table 2:** Comparison of included studies and their method of assessing multiorgan systems in neonatal encephalopathy. The incidence of each organ involvement in the study participants were also noted.

	Neuro	CVS	Resp	GI	Hepatic	Renal	Hem
Perlman et al. (10)	• Abnormal neurological exam (100%) • Abnormal cranial ultrasound (25.7%) Incidence: 37%	• ECG abnormalities (11.4%) • M-mode ECHO (deviated >2 SDs from mean values in normal infants) (8.6%) • Abnormal 2D ECHO with Doppler interrogation (20%) Incidence: 28%	Need for intubation and mechanical ventilation for >48 h Incidence: 100%	Evidence of NEC Incidence: 1 infant developed jejunal perforation without histologic evidence of colitis	N/A	• Azotemia—serum urea >710 mmol/L (11%) • SCr >90 μmol/L after postnatal day 3 (17%) • Oliguria—urine output < 1 ml/kg/hr (transient if present in first 24 h of life; persistent if first 36 h of life) (40%) • First void-urine sample—elevated *β*-2 microglobulin and creatinine concentration (57%) Incidence: 100%	N/A
Martin-Ancel et al. (11)	• Abnormal neurological exam (100%) • Abnormal cranial ultrasound and/or CT scan (8.3%) • Abnormal EEG/clinical seizures (19%) Incidence: 72%	• Heart murmur (21%) • ECG abnormalities (19%) Incidence: 29%	• Need for FiO_2_ > 0.2 for ≥24 h (7%) • Need for mechanical ventilation (19%) Incidence: 26%	• Repeated bloody gastric residuals (22%) or vomiting (6.9%) • Abdominal distension or tenderness Incidence: 29%	N/A	• Oliguria <1 ml/kg/hr for >24 h (53%) • Proteinuria ≥2+ (31%) • Azotemia blood urea nitrogen >7 mmol/L (25%) • Need for parenteral fluid Incidence: 42%	N/A
Hankins et al. (12)	• Clinical evidence of CNS injury • EEG (49%) or neuroimaging abnormalities (20% for head ultrasound, 13% for head CT, and 7% for head MRI) Incidence: 70%	• Need for pressor agents >2 h of life • Elevation of CK-MB isoenzyme (17%) Incidence: 72.8%	N/A	N/A	AST (76%), ALT (35%), LDH (67%) levels 1.5x > upper normal limit Incidence: 80%	• Elevation of SCr ≥1.0 mg% with subsequent return to normal (59%) • Oliguria if persistent >24 h • Persistent hematuria (11%) or proteinuria (9%) Incidence: 72%	• Early thrombocytopenia (≤100 000) (28%) • Increase in nRBC ≥26 per 100 WBCs (41%) Incidence: 54%
Shah et al. (8)	N/A	• Hypotension treated with inotrope for >24 h • ECG evidence of transient myocardial ischemia Incidence: 62%	Need for ventilator support with O_2_ requirement >40% for ≥4 h after birth Incidence: 86%	N/A	AST or ALT >100 IU/L at any time during 1st week after birth Incidence: 85%	• Anuria/Oliguria (<1 ml/kg/hr) for ≥24 h and SCr concentration >100 mmol/L • Anuria/oliguria >36 h • Any SCr >125 mmol/L • Serial SCr values that increased postnatally Incidence: 70%	N/A
Tarcan et al. (13)	• Presence of encephalopathy (95.5%) and/or convulsions (81.8%) • Neuroimaging abnormalities (63.6%)	• Bradycardia (<100 bpm) (22.7%) • Hypotension (13.6%) • Need for vasoactive drugs (dopamine) (18.2%)	• Need for mechanical ventilation (45.5%) or CPAP (36.4%) • Presence of MAS (36.4%)	N/A	Elevated ALT >100 U/L (2x upper normal) Incidence: 39%	• Oliguria (<1 ml/kg/hr) (9.1%) • SCr >1.5 mg/dl (27.3%)	• Polycythemia (4.5%) • Normoblastemia (36.4%) • Thrombocytopenia (<130 000/mm^3^) (59.1%)
Basu et al. (14)	• Abnormal neurological exam • Abnormal aEEG and/or cranial ultrasound	• Abnormal BP, HR, MAP through 12 and 24 h • Need for volume replacement and/or vasopressor in first 24 h	PaO_2_ and PaCO_2_ at 12 h	N/A	Elevated AST, ALT, PT and PTT at 0 and 24 h	• Elevate SCr in next 24 h • Decreased urine output (ml/kg/hr) in first 24 h	Low platelet count at 0 and 24 h
Thorsen et al. (15)	• Abnormal neurological exam (100%) • Presence of seizures at admission (22%) and/or ≥2 days (16%) • Abnormal EEG (57%)	N/A	N/A	N/A	AST (53.5%) and/or ALT (25.4%) > 100 U/L	• SCr >125 mmol/L (8.5%) • Anuria/oliguria (<1 ml/kg/hr) in first 24 h (15%)	N/A
Alsina et al. (16)	N/A	• Troponin T ≥ 0.1 μg/L • Need for vasoactive drugs Incidence: 34% at day 1 for moderate and severe HIE	Need for respiratory support due to other causes than central apnea or pharmacological effect Incidence: 44% at day 1 for moderate and severe HIE	N/A	• GOT or GPT ≥ 100 IU/L • Prothrombin activity ≤60% Incidence: 19% at day 1 for moderate and severe HIE	• SCr ≥1 mg/dl • Diuresis <1 ml/kg/hr • Need for replacement therapy Incidence: 32% at day 1 for moderate and severe HIE	• Leukocyte count <4.5 mm^3^ or >30 mm^3^ • Platelet count <150 mm^3^ • APTT >45 s • No. of platelet or FFP concentrate ≤2 units in 24 h Incidence:% 10 at day 1 for moderate and severe HIE
Michniewicz et al. (17)	N/A	ECHO abnormalities	CXR abnormalities of RDS, MAS, or pneumonia Incidence: 50.9% total	N/A	AST and/or ALT >100 IU/L Incidence: 51.8% in moderate HIE and 93.3% in severe HIE	• SCr >1.5 mg/dl • Had a creatinine increase of 0.3 mg/dl in 24 h • Oliguria (<0.5 ml/kg/hr) Incidence: 7.4% in moderate HIE and 70% in severe HIE	• Thrombocytopenia (<100 000/mm^3^) Incidence: 29.6% in moderate HIE and 70% in severe HIE
Sweetman et al.^a^ (9)	• Abnormal cranial and/or MRI imaging • Clinical seizures and/or with cEEG	• HR <80 bpm • Troponin T > 0.1 ng/ml	• Mechanical ventilation for >72 h • FiO_2_ requirement for >72 h • Pulmonary hypertension	Time to full oral feed ≥6 days	AST and/or ALT >100 IU/L	• Oliguria <1 ml/kg/hr • SCr >100 μmol/L	• Platelets <60 × 10^9^/L • PT >20 s • Fibrinogen <1 g/L
Terzic et al.^a^ (18)	• Presence of seizure—clinically or aEEG confirmed Incidence: 51.6%	Elevated CK, troponin	N/A	N/A	Elevated AST, ALT, LDH	N/A	Elevated INR and APTT
Yan et al. (19)	• Seizures or burst suppression patterns or status epilepticus detected on aEEG or cEEG Incidence: 36.9%	• Need for inotropic support in first 3 days of life (52.9%) • Signs of myocardial dysfunction on ECHO (13.2% LV-EF <55%, 30.9% RV-FAC <29%, 50% TAPSE <7 mm) Incidence: 54.1%	• Need for invasive mechanical ventilation for ≥3 days (44.6%) • Need for extracorporeal membrane oxygenation support • FiO_2_ > 60% at the time of redirection of care • Pulmonary hypertension (29.3%) Incidence: 44.6%	N/A	ALT (30.3%) or AST (64.5%) ≥ 100 IU/L within first 3 days of life Incidence: 64.5%	• SCr—absolute rise ≥0.3 mg/dl or rise ≥1.5× from baseline (9.9%) • Urine output ≤1 ml/kg/hr (15.3%) Incidence: 17.3%	Within first 3 days of life: • Platelet count ≤100 k/μl (38.5%) • INR ≥1.7 (61.3%) Incidence: 68.2%

Neuro, neurological; CVS, cardiovascular; Resp, respiratory; GI, gastrointestinal; Hem, hematological; N/A, not assessed; ECG, electrocardiogram; ECHO, echocardiogram; hr, hour; SD, standard deviation; 2D, two-dimensional; NEC, necrotising enterocolitis; SCr, serum creatinine; CT, computed tomography; (a/c)EEG, (amplitude-integrated/continuous) electroencephalogram; MRI, magnetic resonance imaging; FiO_2_, fraction of inspired oxygen; CNS, central nervous system; CK, creatine kinase; AST, aspartate transaminase; ALT, alanine transaminase; LDH, lactate dehydrogenase; nRBC, nucleated red blood cells; WBC, white blood cells; CPAP, continuous positive airway pressure; MAS, meconium aspiration syndrome; BP, blood pressure; HR, heart rate; MAP, mean arterial pressure; PaO_2_, partial pressure of oxygen; PaCO_2_, partial pressure of carbon dioxide; PT, prothrombin time; PTT, partial thromboplastin time; GOT, glutamic oxaloacetic transaminase; GPT, glutamic pyruvic transaminase; APTT, activated partial thromboplastin time; FFP, fresh frozen plasma; CXR, chest x-ray; RDS, respiratory distress syndrome; INR, international normalized ratio; LV-EF, left-ventricular ejection fraction; RV-FAC, right ventricular fractional area change; TAPSE, tricuspid annular plane systolic excursion.

^a^ Incidence values of organ systems could not be extracted due to different outcome measurements or not stated by authors.

Organ dysfunction may have been initiated at any stage from conception to delivery and early childhood and is initially reliant on maternal-placental-fetal triad function. In addition, there are lifelong effects of the exposome comprising toxic stressors affecting neurodevelopment initially as gene-environment interactions in the first 1,000 days of life when 80% of neuronal connections are made (25, 26).

### Heterogeneity in scoring system parameters may complicate consensus development

Assessment of neurological dysfunction varied slightly across studies, though most used some combination of neuroimaging or EEG recording with clinical parameters. Neurological dysfunction was not included as a parameter in three studies, and one study cited its consideration as a baseline patient characteristic as opposed to a parameter of organ dysfunction as the reason for this exclusion (17). The question remains as to whether neurological dysfunction should be included in MOD scoring systems and further study is required relating to its value in the prediction of morbidity and mortality in the context of MOD despite its known validity in assessment in single-organ-system scores such as the Sarnat score (6).

Cardiovascular dysfunction was also included in most studies, however, there was significant heterogeneity in the definition of cardiovascular dysfunction across studies. Several studies included the requirement of vasoactive medications or inotropic support as indicators of cardiovascular dysfunction. Evidence suggests that in NE, inotrope requirement is associated with increased risk of death or MRI-detected brain injury (27). Others relied on echocardiographic evidence of cardiac dysfunction (17), clinical parameters such as blood pressure or heart rate (9, 13), ECG features indicative of myocardial ischemia (8), or biochemical tests such as troponin T or creatine kinase (16, 18). Cardiovascular dysfunction is complex following hypoxia-ischemia (28), and this may be a contributing factor to the variation seen across studies in the definition of cardiovascular dysfunction for the purpose of scoring systems. This variation complicates the validation of any one of the scoring systems.

Respiratory system dysfunction definitions were considerably less variable across studies in which respiratory dysfunction was included. Most studies defined respiratory dysfunction by the requirement of some level of ventilatory or other respiratory support. Some studies included pulmonary hypertension in their definition of respiratory dysfunction, however, pulmonary hypertension was shown to be the only measure of organ dysfunction in one study not associated with death or moderate to severe brain injury (19). Gastrointestinal dysfunction was not included in nine of the twelve studies evaluated. Those that did include gastrointestinal dysfunction defined it mainly by various clinical parameters. The reason for the omission of gastrointestinal dysfunction from most studies is unclear.

Hepatic dysfunction, however, was almost universally included in scoring systems evaluated. It has been shown that increasing severity of HIE correlates with serum levels of ALT and AST (29). Most studies included derangements in ALT or AST in their definition of hepatic dysfunction for the purpose of a MOD scoring system. Other parameters such as LDH, prothrombin activity, GOT, and GPT were also included in some studies (12, 16) in the definition of hepatic dysfunction.

Renal dysfunction was also included in all but one of the studies. Renal dysfunction was defined by most scoring systems using either oliguria or anuria, or elevations in serum creatinine levels. Acute kidney injury in neonates is typically defined by the KDIGO guidelines and has been associated with HIE outcomes (30). O’Dea and colleagues note, however, that serum creatinine may not be an appropriate biomarker of acute kidney injury in neonates given the delay to its peak and other limitations (7). Electrolyte disturbance is also a prominent feature following birth asphyxia, where hyponatremia and hyperkalemia are directly proportional to the severity of asphyxia (31). Electrolyte disturbance was assessed only by Alsina and colleagues in their MOD scoring system where it was identified as one of the most affected systems (16), however, most studies did not include electrolyte disturbance in their criteria for defining renal dysfunction. Given the implications of fluid balance on neurological outcomes for neonates with HIE (19, 32), and the complexity associated with fluid management in these patients further complicated by renal dysfunction, it should be expected that any consensus MOD scoring system will include renal dysfunction as a key parameter.

Hematological dysfunction is well-documented in NE. HIE is associated with significant coagulopathy and this has implications for treatment strategies especially relating to the transfusion of blood products (33). Thus, the inclusion of hematological dysfunction in the majority of studies is unsurprising given the obvious implications of severe coagulopathy on long term outcomes. Thrombocytopenia was used in the definition of hematological dysfunction in seven of the eight studies which included hematological dysfunction in their scoring of MOD. Other studies included PT, APTT, and INR in their definition (9, 16, 19) however, this may relate more to dysfunction of hepatic synthetic functions. This relationship between hepatic and hematological dysfunction is one example of a possible dispute which may further complicate the development of a comprehensive and easily-applied MOD scoring system, as alluded to by Tarcan and others (13). Hankins et al. and Tarcan et al. were the only studies that included red blood cell count as parameters of hematological dysfunction. Hankins and colleagues specifically used nucleated red blood cell count, given that there is increase in production after a period of fetal hypoxia (34).

### Therapeutic hypothermia also has multiorgan effects

TH is the standard of care for moderate to severe NE (35). The neonate is cooled between 33°C to 35°C, usually within six hours of birth, and typically with a cooling blanket (36, 37). Cooling lasts for 72 h followed by rewarming at a rate of 0.5°C per hour (37). There is strong evidence that therapeutic cooling is neuroprotective, reducing mortality and neurological morbidity (38).

There is a scarcity of studies assessing the effect of TH on MOD in the context of NE. Some evidence suggests that TH is beneficial in reducing the severity of MOD. For instance, TH was found to reduce blood levels of cardiac troponin 1, a marker released by injured cardiomyocytes, when compared to neonates who didn’t undergo TH (39). TH has also been found to have an anti-inflammatory effect, producing a delay in the rise of C-reactive protein (CRP) and reducing the peak CRP response, white cell count, and platelet count (40).

However, there is evidence that TH may exacerbate MOD. A meta-analysis of seven randomized controlled trials (RCTs) found that TH increased the rates of cardiac arrhythmia during the intervention (41). TH is also associated with thrombocytopenia, although it doesn’t appear to increase the risk of haemorrhage (42). In addition, TH is associated with higher rates of bradycardia, pressor requirement, metabolic acidosis, hematuria, and seizures (43). Thus, the precise impact TH has on MOD needs further elucidation.

MOD during TH may be a key consideration in MOD scoring system evaluation. The identified studies investigating MOD in NE collected data at various and sometimes unspecified time points in the days following birth, some of which were during TH treatment. Five studies did not measure any effects of TH as the treatment was not the standardized care at the time (8, 10–13). Alsina et al. and Michniewicz et al. measured the parameters before TH whilst five studies measured after the application of TH (16, 17). This may explain the negative findings in the neurodevelopmental outcome in Shah et al. compared to Sweetman et al. that also used the Bayley score for the same outcome (2, 8, 9). However, only 39 out of 85 of patients from Sweetman et al. underwent TH but there was no report of difference between the outcomes with or without TH (9). Moderate hypothermia may be associated with some level of MOD, and this may have to be considered in the development of a scoring system, specifically relating to the timing of MOD parameter measurement. This MOD during TH is not significantly different between different cooling methods (44). The cooling status of patients in the studies evaluated in this review is variable and thus, further investigation of the effect of TH on MOD and the measurement of some clinical parameters relative to the timing of hypothermia initiation is necessary.

### Limitations

To the authors’ knowledge, this is the first systematic review that has evaluated the different systems used for assessing MOD in NE. As such, a quantitative appraisal was challenging to execute. There is heterogeneity in the design of studies included, though given the scarcity of specific research in this area, wider inclusion criteria had to be adopted. Variability existed in the definition of NE or HIE, biomarkers used to define perinatal asphyxia, gestational age, clinical parameters, and cooling status of included patients. Furthermore, there were significant discrepancies in the follow-up time of each study, which may affect the validity of certain outcome measures discussed. Heterogeneity in outcome assessment between studies also limited the comparison of MOD scoring systems. TH may be a significant confounder since there were differences in the introduction and timing of TH on the patients in the studies. Moreover, it was only implemented in clinical practice after the year 2005 so some of the included studies would not have TH in their management (21, 35). This review was also inherently limited by the number of databases searched for relevant literature and the inclusion of only papers available in English.

There are limitations to the systematic review methodology as there is heterogeneity of data and methods in individual studies and there is a potential for outdated results. In the future newer methods such as causal inference of observational studies would augment the information from randomized studies (45, 46). This paper is an initial step towards the development of a consensus evidence-based evaluation of organ dysfunction in neonates with encephalopathy similar to the recent PODIUM network in Pediatric sepsis (47). This would involve a Delphi questionnaire to the international community including all stakeholders and families in HIC and LMIC to define the core measures of organ dysfunction (4, 48). Following the development of larger datasets based on consensus data points this an organ dysfunction score could be developed to correlate with outcome and ultimately allow individualized targeted follow up of specific organ dysfunction. These studies did not define aetiology and therefore we could not distinguish the differences between them (3, 49, 50). Defining aetiology is important for future consensus and refining therapies beyond therapeutic hypothermia in NE and in future studies would ideally be part of standardized reporting. Again, this paper highlights these deficiencies in reporting. Establishing etiology is crucial in NE due to the multiple causes such as infection, placental dysfunction/inflammation, genetic, metabolic and other causes that will allow refinement and individualization of clinical management. Redline et al. describe the limitations of the Amsterdam criteria to give details of etiopathogenesis (51).

### Future perspectives

There needs to be consistency in future studies assessing MOD in NE, particularly on the inclusion criteria of participants with NE and the variables used in measuring the organ systems. Available guidelines for defining organ function can be used to overcome this challenge such as the Kidney Disease: Improving Global Outcomes (KDIGO) guidelines for acute kidney injury (52). It would also be useful to measure the severity of NE before commencing TH to minimize its potential confounding effects. The clinical multiorgan scoring systems would have potentially improved diagnostic power with the addition inflammatory or neuronal biomarkers and the use of AI in larger multinational datasets. Ongoing work in the Newborn Brain Society aims to standardize Neonatal Encephalopathy registry datasets which will be crucial to improve prognostication in NE (53).

An ideal scoring system should be universally applicable, specifically in low-resource settings where neonatal mortality rates are higher. Measurements such as abnormal neurological exam including the presence of seizures, abnormal vital signs, need for respiratory support, elevated liver enzyme levels, oliguria or elevated serum creatinine, and low platelet count should be considered in the MOD scoring system as they would be accessible in these settings since they would be routinely used in baseline measurements for many indications. This systematic review would be an ideal supplement, besides repeating the existing systems for validation and the Delphi method (54), to form a standardized scoring system.

## Conclusion

This review aimed to assess the current literature on MOD scoring systems for NE. Of the 12 included studies, there was substantial heterogeneity between the scoring systems. Variability was found across three main areas: (1) the inclusion criteria, (2) the organs assessed, and (3) the methods of system assessment, limiting the comparison of conclusions that can be made between studies. There was greater consensus in reporting for the renal, hepatic, respiratory, neurological, hematological, and cardiac systems and therefore, should be considered in an MOD scoring system. However, improved assessment and inclusion of neurological and cardiac system outcomes are warranted. Of note, scores that included more systems improved the accuracy in predicting adverse events and mortality. The existing scoring systems should be repeated to further validate the parameters. Additionally, more cost-effective and accessible variables should be included in the scoring systems.

## Data Availability

The original contributions presented in the study are included in the article/Supplementary Material, further inquiries can be directed to the corresponding author.
